# Missense Variants of Uncertain Significance (VUS) Altering the Phosphorylation Patterns of BRCA1 and BRCA2

**DOI:** 10.1371/journal.pone.0062468

**Published:** 2013-05-21

**Authors:** Eric Tram, Sevtap Savas, Hilmi Ozcelik

**Affiliations:** 1 Fred A. Litwin Centre for Cancer Genetics, Samuel Lunenfeld Research Institute, Toronto, Ontario, Canada; 2 Department of Pathology and Laboratory Medicine, Mount Sinai Hospital, Toronto, Ontario, Canada; 3 Department of Laboratory Medicine and Pathobiology, University of Toronto, Toronto, Ontario, Canada; Ohio State University Medical Center, United States of America

## Abstract

Mutations in *BRCA1* and *BRCA2* are responsible for a large proportion of breast-ovarian cancer families. Protein-truncating mutations have been effectively used in the clinical management of familial breast cancer due to their deleterious impact on protein function. However, the majority of missense variants identified throughout the genes continue to pose an obstacle for predictive informative testing due to low frequency and lack of information on how they affect BRCA1/2 function. Phosphorylation of BRCA1 and BRCA2 play an important role in their function as regulators of DNA repair, transcription and cell cycle in response to DNA damage but whether missense variants of uncertain significance (VUS) are able to disrupt this important process is not known. Here we employed a novel approach using NetworKIN which predicts *in vivo* kinase-substrate relationship, and evolutionary conservation algorithms SIFT, PolyPhen and Align-GVGD. We evaluated whether 191 *BRCA1* and 43 *BRCA2* VUS from the Breast Cancer Information Core (BIC) database can functionally alter the consensus phosphorylation motifs and abolish kinase recognition and binding to sites known to be phosphorylated *in vivo*. Our results show that 13.09% (25/191) *BRCA1* and 13.95% (6/43) *BRCA2* VUS altered the phosphorylation of BRCA1 and BRCA2. We highlight six *BRCA1* (K309T, S632N, S1143F, Q1144H, Q1281P, S1542C) and three *BRCA2* (S196I, T207A, P3292L) VUS as potentially clinically significant. These occurred rarely (n<2 in BIC), mutated evolutionarily conserved residues and abolished kinase binding to motifs established in the literature involved in DNA repair, cell cycle regulation, transcription or response to DNA damage. Additionally *in vivo* phosphorylation sites identified via through-put methods are also affected by VUS and are attractive targets for studying their biological and functional significance. We propose that rare VUS affecting phosphorylation may be a novel and important mechanism for which BRCA1 and BRCA2 functions are disrupted in breast cancer.

## Introduction

Rare germline mutations of *BRCA1* and *BRCA2* predispose carriers to early-onset familial breast or ovarian cancers [1]–[3]. These genes can account for half of breast and/or ovarian familial cancer aggregates (whereas the remaining families receive inconclusive results) and are responsible for about 5–10% of all breast cancer cases and 10–15% of ovarian cancers in the general population [4], [5]. Clinically informative results from *BRCA* screening have been mostly derived from protein-truncating mutations presenting as indels, nonsense codons and splice variants as well as large genomic rearrangements [3], [6], [7]. Such mutations have very apparent impacts on the normal protein function and have been widely utilized in the clinical management of familial breast and ovarian cancers. However, further analysis of a significant number of *BRCA1* and *BRCA2* missense variants of uncertain significance (VUS) continue to pose an important obstacle to the clinical management of a considerable portion of familial breast cancer probands and families who carry such VUS.

Previously, the need to characterize missense variants to provide risk assessment to individuals from high-risk families led to development of several approaches in classifying VUS. These include integrating interspecies sequence variation [8]–[10], functional analysis to uncover the consequences of VUS on protein function [11]–[14], genetic assessment approaches including pedigree analysis [15], likelihood models [16], structural-based approaches to model the effect of amino acid substitution [17], [18] and transcriptional activity assays [19]. These studies have provided important information into the clinical significance of *BRCA* mutations.

Phosphorylation is an important post-translational modification that occurs at specific serine, tyrosine and threonine residues within protein sequences [20]. The phosphorylated residue is surrounded by a kinase interaction/recognition motif that is typically comprised of 7–12 amino acids [21] and that kinase specificity is determined by the identity of these residues [22], [23].

Our studies have previously suggested that missense VUS and commonly occurring single nucleotide polymorphisms (SNPs) altering phosphorylation patterns of cell cycle and DNA repair proteins may contribute to human cancer risk [24], [25] and our preliminary analysis showed that many of the missense variants in BIC are found within the consensus motifs of sites known to be phosphorylated *in vivo.* Despite this wealth of information, the potential functional impact of these rare VUS remains uncharacterized. In the present study, our goal is evaluate the potential consequences of missense VUS on kinase recognition and phosphorylation of BRCA1 and BRCA2 proteins. Accordingly, we have utilized the web-based algorithm NetworKIN 2.0 [26] and selectively tested the missense VUS listed in the BIC database that are located within 10 amino acids around the experimentally verified and biologically characterized phosphorylation sites as well as residues identified via high-throughput methods to be phosphorylated *in vivo*. Here, we analyzed 191 *BRCA1* and 43 *BRCA2* missense VUSs, which have the potential to interfere with the phosphorylation process via abolishing or creating phosphorylation sites on BRCA1 and BRCA2.

## Methods

### Selection of *in vivo* Phosphorylation motifs for analysis

**Figure 1:**
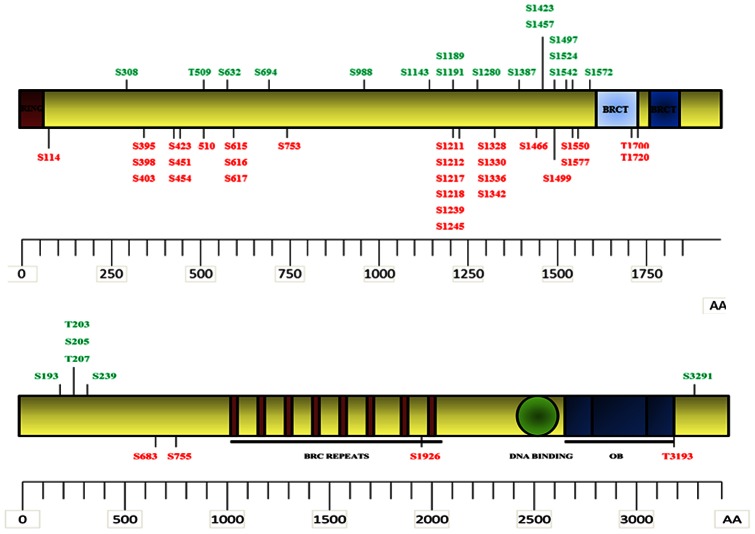
**a.** Summary of phosphorylation sites studied in BRCA1. Residues in green represent *in vivo* phosphorylation sites have been biologically characterized in the literature. Residues in red represent *in vivo* phosphorylation sites identified via throughput methods where biological functions have not yet been determined. **b.** Summary of phosphorylation sites studied in BRCA2. Residues in green represent *in vivo* phosphorylation sites that have been biologically characterized in the literature. Residues in red represent *in vivo* phosphorylation sites identified via throughput methods where biological functions have not yet been determined.

A comprehensive list of known phosphorylation sites of BRCA1 and BRCA2 was obtained from the curated databases PhosphositePlus [27] and Phospho. ELM [28] as of August 2012. We evaluated *BRCA1* and *BRCA2* missense variations' effect in relation to 44 and 11 phosphorylation sites reported in humans, respectively (Figure 1a, b). Based on the curated databases, all sites selected were reported to be phosphorylated *in vivo* and reported in the literature. Kinase binding and biological significance of the phosphorylation on protein function had been demonstrated for sixteen sites in BRCA1 and six sites in BRCA2. Accordingly, these experimentally characterized sites are denoted “biologically characterized” in this manuscript. The remaining sites were previously identified as phosphorylated *in vivo* using high-throughput methods (e.g. Mass spectrometry) where a definitive biological significance in protein function has not yet been shown and are designated as “biologically uncharacterized” in this manuscript.

### Missense VUS from the Breast Cancer Information Core Database

The National Institute of Health (NIH)'s Breast Cancer Information Core (BIC) database (http://research.nhgri.nih.gov/bic/) contains 11 types of genetic variations. These genetic variations are identified by studying the tumor DNA samples and may therefore be either inherited or somatic variations. Using the most up-to-date version of the BIC database as of August 2012, 591 *BRCA1* and 883 *BRCA2* missense VUSs were retrieved. Only VUS located in or within a 10 amino acids sequence upstream and downstream of a phosphorylation site were selected for analysis. A total of 191/591 *BRCA1* and 43/883 *BRCA2* missense variants located in or near a kinase recognition motif were included in this study.

### NetworKIN analysis of VUS on BRCA1 and BRCA2 phosphorylation

BRCA1 (Genbank P38393) and BRCA2 (Genbank P51587) protein sequences were queried by the NetworKIN Beta 2.0 algorithm (http://networkin.info/version_2_0/search.php) [26], an improved version of the NetworKIN algorithm featuring more kinases. The NetworKIN tool is designed to predict *in vivo* kinase-substrate relations [26]. It remains up to date with the most current human phosphoproteome information derived from Phospho.ELM and PhosphoSite databases and these sites are compared with sequence motifs predicted using the Scansite [29] and NetphosK [30] programs to predict the kinase families that potentially bind and phosphorylate such sequences. The algorithm takes into account also the biological context of a kinase through the use of probabilistic functional associations from the STRING database [31].

The BRCA1 or BRCA2 protein sequences carrying each VUS substitution was queried by NetworKIN and the output matched to predictions made for the wild-type protein sequence. VUS which result in abolishing kinase binding at the phosphorylation motif or create a site at the altered residue are included in this report. Furthermore only the predictions for kinase-phosphorylation motif interactions with a NetworKIN score ≥5 were considered reliable (Dr. Rune Linding, personal communication). In cases where multiple kinases are predicted to bind a phosphorylation site with a NetworKIN score ≥5 we arbitrarily assumed the abolition of 80% or more of the kinase binding to be the equivalent to the complete abolition of a phosphorylation motif.

### Evolutionary conservation analyses

To determine whether the missense VUSs substitute functionally critical residues we have investigated their evolutionary conservation status using: (1) Sorting Intolerant From Tolerant (SIFT; http://blocks.fhcrc.org/sift/SIFT.html). SIFT (V.2) is a multiple sequence alignment tool that was developed based on the idea that amino acids which play an important role tends to be conserved in the protein family, so changes at these sites would be deleterious to protein function [32]. SIFT analysis was performed using algorithms to find homologous sequences from database SWISS-PORT version 51.3 and TrEMBL 34.3, and selecting median conservation sequence score 3.00. Predictions out of the accepted median sequence conservation score of 2.75–3.25 were also considered not reliable and thus were considered “not informative”. (2) PolyPhen (Phenotypic Polymorphism); (http://genetics.bwh.harvard.edu/pph2/). PolyPhen-2 v.2.2.2r398 predicts the impact of an amino acid substitution on the structure and function of a human protein [33]. (3) Align-grantham variation grantham deviation (GVGD) specific weighted evolutionary conservation analysis was carried out for BRCA1 and BRCA2 (http://http://agvgd.iarc.fr/agvgd_input.php) to determine the A-GVGD class of each variants presented [10]. A-GVGD uses the biochemical characteristics of amino acids together with protein sequence alignments of multiple species to determine whether a missense mutation could be neutral or deleterious to protein function. A-GVGD was used with all default settings. Library alignments for BRCA1 and BRCA2 were selected and analysis was performed using the longest evolutionary depth (Human to Sea Urchin).

Although PolyPhen also uses other assessment criteria such as protein 3-dimensional structure, both SIFT and PolyPhen use alignment of similar proteins to determine whether an amino acid is conserved and whether its substitution by a VUS has potential functional consequences. To standardize the predictions made by these two tools, we have annotated the “affecting protein function” prediction of SIFT and both the “probably damaging” and “possibly damaging” predictions of PolyPhen as “damaging” in this report. Similarly, the “tolerated” prediction of SIFT and the “benign” prediction of PolyPhen are collectively annotated as “benign”. For any predictions that include a “damaging” and “benign/tolerated” output of either program, we have annotated such VUS as “likely damaging”.

## Results

### Study design and overall findings

Using NetworKIN Beta 2.0, we investigated the impact of 191 *BRCA1* and 43 *BRCA2* missense VUS found within or around 44 BRCA1 and 11 BRCA2 phosphorylation sites, respectively (Figure 1a, b, Tables S1 & S2 in File S1). Our analysis indicated that 13.09% (25/191) *BRCA1* and 13.95% (6/43) *BRCA2* VUSs impact an existing phosphorylation site, and/or create a new site at the altered residue (Table 1, 2). Specifically six *BRCA1* and three *BRCA2* VUS resulted in deleterious NetworKIN predictions at experimentally and biologically characterized phosphorylation sites while nineteen *BRCA1* and three *BRCA2* VUS similarly affected biologically uncharacterized phosphorylated sites. In cases where NetworKIN predictions of kinases differ from those identified experimentally, we found in most cases the prediction fell within the same family of protein kinases. The Leiden Open Variation Database (LOVD v.2.0 build 35; http://chromium.liacs.nl/LOVD2/cancer/home.php) was accessed and VUS highlighted by this study and included in previous studies are summarized in Table S3 and S4 in File S1.

**Table 1:** NetworKIN analysis of BIC VUSs affecting biologically characterized phosphorylation motifs in BRCA1 and BRCA2.

Protein	Mutation^a^	Nucleotide Change^b^	SNP Id^c^	Exon	BIC Freq^d^	NetworKIN Results^e^	SIFT/Polyphen/A-GVGD	Biological Significance of Affected Phosphorylation Motif
BRCA1	p.K309T	c.926A>C	rs80356877	11A	1	T309 abolishes STK6 binding at S308 in FCNKSKQPGL and creates ATM binding to T309 in FCNKSTQPGL	Damaging (C0)	Loss of STK6 binding decreases G(2) to M transition of the cell cycle in cells [50]
**BRCA1**	**p.S632N**	**c.1895G>A**	**rs80356983**	**11B**	**1**	**N632 abolishesCDK2** **binding to S632 in VSRNLSPPNCT**	**Likely Damaging (C0)**	**S632A affects BRCA1-dependent regulation of transcription ** [**48**]
BRCA1	p.P633T	c.1897C>A	N/A	11B	1	T633 abolishes CDK2 binding to S632 in VSRNLSPPNCT and creates CDK2 binding to T633 in SRNLSTPNCT	Likely Damaging (C0)	S632A affects BRCA1-dependent regulation of transcription [48]
BRCA1	p.P633S	c.1897C>T	rs80356902	11B	1	S633 abolishes CDK2 binding to S632 in SRNLSPPNCT and creates CDK2, MAPK14, MAPK13, MAPK11, MAPK10, MAPK9, MAPK8 binding to S633 in SRNLSSPNCT	Likely Damaging (C0)	S632A affects BRCA1-dependent regulation of transcription [48]
**BRCA1**	**p.S1143F**	**c.3428C>T**	**rs80357434**	**11D**	**1**	**F1143 abolishes ATM binding to S1143 in SSHASQVCSE**	**Likely Damaging (C0)**	**S1143 inactivation reduces intracellular localization of BRCA1 into MMTS-induced loci ** [**46**]
BRCA1	p.Q1144H	c.3432G>T	rs80356922	11D	1	H1144 abolishes ATM binding to S1143 in SSHASQVCSE	Likely Damaging (C0)	S1143 inactivation reduces intracellular localization of BRCA1 into MMTS-induced loci [46]
BRCA1	p.Q1281P	c.3842A>C	rs80357483	11D	2	F1281 abolishes ATM binding to S1280 in LAKASQEHHL	Damaging (C0)	S1280 inactivation reduces intracellular localization of BRCA1 into MMTS-induced loci [72]
**BRCA1**	**p.S1542C**	**c.4625C>G**	**rs41293457**	**15**	**2**	**C1542 abolishes CSNK2A2, CK2A1 binding to S1542 in QLEESGPHDL**	**Likely Damaging (C0)**	**S1542 phosphorylated by ATM and possibly involved in response to DNA double-strand breaks produced by ionizing radiation ** [**49**]
BRCA2	p.S196I	c.587G>T	rs80358818	7	1	I1961 abolishes TGFBR2, ACVR2B binding to S193 in VDPDMSWSSS	Damaging (C65)	Phosphorylation of S193 regulates BRCA2 interaction with p300/CBP-associated factor (P/CAF) [56]
**BRCA2**	**p.T207A**	**c.619A>G**	**rs80358858**	**7**	**2**	**A207 abolishes NEK2 binding to T207 in TLSSTVLIVR**	**Damaging (C55)**	**Phosphorylation of T207 regulates BRCA2 interaction with p300/CBP-associated factor (P/CAF) ** [**56**]
BRCA2	p.P3292L	c.9865C>T	rs56121817	27	7	P3292 abolishes CDK2, MAPK11, MAPK13, MAPK14 binding to S3291 at CTFVSPAAQK	Damaging (C0)	S3291phosphorylation necessary for recombinatory repair [44], [45]

In **bold** are BRCA1 mutations that directly mutate an experimentally identified phosphorylation site. ^a^The position and change at the amino acids specified by the missense variant is as reported in the BIC database. ^b^The nucleotide change conforms to the HGVS nomenclature. ^c^SNP IDs correspond to the dbSNP database [73] SNP identifiers. ^d^Frequency represents the number of times reported in the BIC database. ^e^The ten-residue long biologically uncharacterized kinase recognition motifs are shown. The biologically uncharacterized Serine (S), and threonine (T) residues shown to be phosphorylated by NetworKIN are underlined.

**Table 2:** NetworKIN analysis of VUS affecting biologically uncharacterized phosphorylation sites in BRCA1 and BRCA2.

Protein	Mutation^a^	Nucleotide Change^b^	SNP Id^c^	Exon	BIC Freq^d^	NetworKIN Results^e^	SIFT/Polyphen/A-GVGD	Biological pathway of Phosphorylation site
BRCA1	**p.S403F**	**c.1208C>T**	**rs80356934**	**11A**	**1**	**F403 abolishes CK2A1 and CSNK2A1 binding to S403 in HDGESESNAK**	**Benign (C0)**	**Cell cycle regulation by protein phosphorylation by cyclin-dependent kinases (CDK) ** [**65**]
BRCA1	p.N417S	c.1250A>G	rs80357113	11A	2	S417 creates CK2A1, CSNK2A1 binding to S417 in VLDVLNEVDE	Benign (C0)	
BRCA1	p.D420Y	c.1258G>T	rs80357488	11A	3	Y420 creates IGF1R, INSR binding to Y420 in VLNEVYEYSG	Damaging (C15)	
BRCA1	**p.S454N**	**c.1361G>A**	**rs80357181**	**11A**	**1**	**N454 abolishes CK2A1 and CSNK2A1 binding to S454 in KSVESNIEDK**	**Benign (C0)**	**DNA damage response following ionizing radiation (IR) ** [**66**]
BRCA1	p.N609S	c.1826A>G	rs80357326	11A	1	S609 creates PRKDC binding to S609 in APKKSRLRRK	Likely Damaging (C0)	
BRCA1	p.R612G	c.1834A>G	rs80357245	11A	1	G623 abolishes RPS6KB1 binding to S615 in LRRKSSTRHI	Likely Damaging (C0)	Cell growth, proliferation via Akt-RSK-S6 signaling network [42]
BRCA1	p.D749Y	c.2245G>T	rs80357114	11B	1	Y749 abolishes CK2A1 and CSNK2A1 binding to S753 in KDLMLSGERVL	Damaging (C0)	Phosphorylation site occupancy during Mitosis [65], [67]
BRCA1	p.G1201S	c.3601G>A	rs55725337	11D	3	S1201 creates NEK2, PRKCD, PRKCI, PRKCQ, PRKCZ, PRKCA, PRKCG binding at HLAQSYRRGA	Benign (C0)	
BRCA1	p.E1214K	c.3655G>A	N/A	11D	9	K1214 abolishes CK2A1 and CSNK2A1 binding to S1211* in AKKLESSEEN and S1212 in KKLESSEENL	Damaging (C0)	
**BRCA1**	**p.S1217P**	**c.3649T>C**	**N/A**	**11D**	**1**	**P1217 abolishes CK2A1 and CSNK2A1 binding** **to S1218 in EENLSSEDEE**	**Damaging (C65)**	
**BRCA1**	**p.S1218C**	**c.3652A>T**	**rs80356894**	**11D**	**2**	**C1218 abolishes CSNK2A2, CK2A1binding to S1218 in EENLSSEDEEL**	**Damaging (C25)**	**Phosphorylation site occupancy during Mitosis ** **[65]**, **[67]**
BRCA1	p.R1507T	c.4520G>C	rs80357470	15	2	T1507 creates TGFBR2, ACVR2B binding at T1507 in SLDDTWYMHS	Likely Damaging (C0)	
BRCA1	**p.T1550I**	**c.4649C>T**	**rs80357076**	**15**	**3**	**I1550 abolishes NEK2 binding to T1550 in HDLTETSYLP**	**Benign (C0)**	**Phosphorylation sites in cellular proteins sensitive to rapamycin ** [**74**]
BRCA1	**p.S1577P**	**c.4729T>C**	**rs80356909**	**16**	**1**	**P1577 abolishes CSNK2A2, CK2A1 binding to S1577 in SDDPESDPSE**	**Likely Damaging (C0)**	**Phosphorylation site occupancy during mitosis ** [**67**]
BRCA1	p.A1584S	c.4750G>T	rs80357070	16	1	S1584 creates CDK2, MAPK8, MAPK10, MAPK9, MAPK14, MAPK11, MAPK13 binding at S1584 in PSEDRSPESA	Benign (C0)	
BRCA1	p.F1695L	c.5085T>A	rs80357837	18	1	L1695 abolishes TGFBR2, ACVR2B, PRKCD, PRKC, PRKCQ, PRKCZ, PRKCA, PRKCG, MST2 binding at T1700 in FVCERTLKYF	Likely Damaging (C0)	DNA damage response [55]
BRCA1	p.R1699L	c.5096G>T	rs41293459	18	1	L1699 abolishes PRKCD, PRKC, PRKCQ, PRKCZ, PRKCA, PRKCG, MST2 binding at T1700 in VCERTLKYFLG	Damaging (C65)	DNA damage response [55]
BRCA1	p.R1699W	c.5095C>T	rs55770810	18	13	W1699 abolishes PRKCD, PRKC, PRKCQ, PRKCZ, PRKCA, PRKCG, MST2 binding at T1700 in VCERTLKYFL	Damaging (C65)	DNA damage response [55]
BRCA1	**p.T1720A**	**c.5158A>G**	**rs56195342**	**19**	**15**	**A1720 abolishes ATM binding to T1720 in YFWVTQSIKE**	**Likely Damaging (C0)**	**DNA damage response ** [**55**]
BRCA2	p.D1923A*	c.5768A>C	rs45491005	11E	9	A1923 abolishes CSNK2A2, CK2A1 binding to S1926 in ADIQSEEILQ	Damaging (C0)	General Mass Spec screen [61]
BRCA2	p.D1923V*	c.5768A>T	rs45491005	11E	1	V1923 abolishes CSNK2A2, CK2A1 binding to S1926 in ADIQSEEILQ	Damaging (C0)	General Mass Spec screen [61]
BRCA2	p.P3194Q	c.9581C>A	rs28897760	26	6	Q3194 abolishes CDK2 binding and creates ATM binding to T3193 in PKWSTPTKDC	Damaging (C0)	General Mass Spec screen [61]

In **bold** are BRCA1 mutations that fall within an experimentally identified but biologically uncharacterized phosphorylation site. ^a^The position and change at the amino acids specified by the missense variant is as reported in the BIC database. ^b^The nucleotide change conforms to the HGVS nomenclature. ^c^SNP IDs correspond to the dbSNP database [73] SNP identifiers. ^d^Frequency represents the number of times reported in the BIC database. ^e^The ten-residue long biologically uncharacterized kinase recognition motifs are shown. The biologically uncharacterized Serine (S), and threonine (T) residues shown to be phosphorylated by NetworKIN are underlined. * Sites that retained a score but was considered to be “abolished” due to score falling below 5 with the presence of the VUS.

### VUS impacting biologically characterized phosphorylation sites

Six *BRCA1* VUS (K309T, S632N, S1143F, Q1144H, Q1281P, S1542C) were predicted to affect the phosphorylation status of BRCA1 by abolishing kinase interaction at experimentally verified sites Ser^308^, Ser^632^, Ser^1143^, Ser^1280^, and Ser^1542^ (Table 1). Three of the aforementioned substitutions (S632N, S1143F, S1542C) directly altered the Serine residue of the phosphorylated sites Ser^632^, Ser^1143^, and Ser^1542^, resulting in the complete abolition of their respective kinase binding without creating new kinase binding. In *BRCA2*, S196I and P3292L VUS altered the consensus kinase motif for Ser^193^ and the sequence for CDK2 binding for Ser^3291^, respectively and T207A directly altered the phosphorylated Threonine residue and completely abolished kinase binding at Thr^207^ (Table 1).

### VUS impacting biologically uncharacterized phosphorylation sites

A total of nineteen *BRCA1* and three *BRCA2* VUS were found to affect biologically uncharacterized phosphorylation sites. These sites were shown to be phosphorylated in *in vivo* experiments; however their potential roles on protein and subsequent cellular function have not been investigated yet. Affecting *BRCA1* were twelve VUS associated with the complete abolition of kinase binding motif without creating binding sites for kinases. These VUS included the S1217P, S1218C, T1550I, S1577P, and T1720A, which removed the phosphorylated residues at Ser^1217^, Ser^1218^, Thr^1550^, Ser^1577^, and Thr^1720^, respectively (Table 2). Additionally, seven VUS substituted the wild-type residue with Y, S or T resulting in the creation of putative kinase binding site at the altered residue. In *BRCA2*, three VUS, D1923A, D1923V and P3194Q, were all predicted to abolish kinase binding while none was predicted to create a new kinase binding site (Table 2).

### Evolutionary conservation of VUS

**Figure 2:**
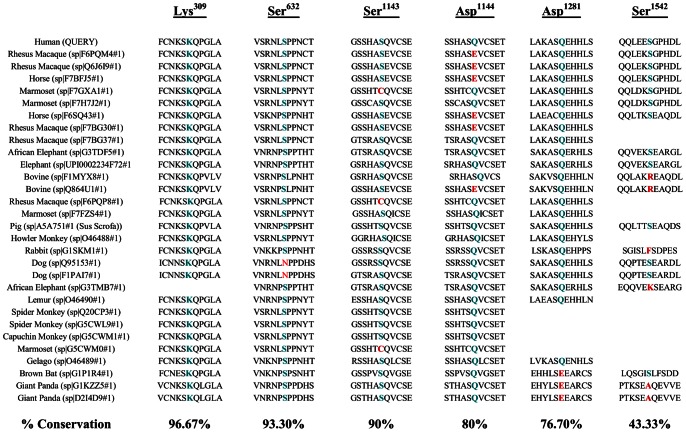
Multiple sequence alignment demonstrating evolutionary conservation of the six biologically characterized phosphorylated BRCA1 residues affected by missense variants of unknown clinical significance.

**Figure 3:**
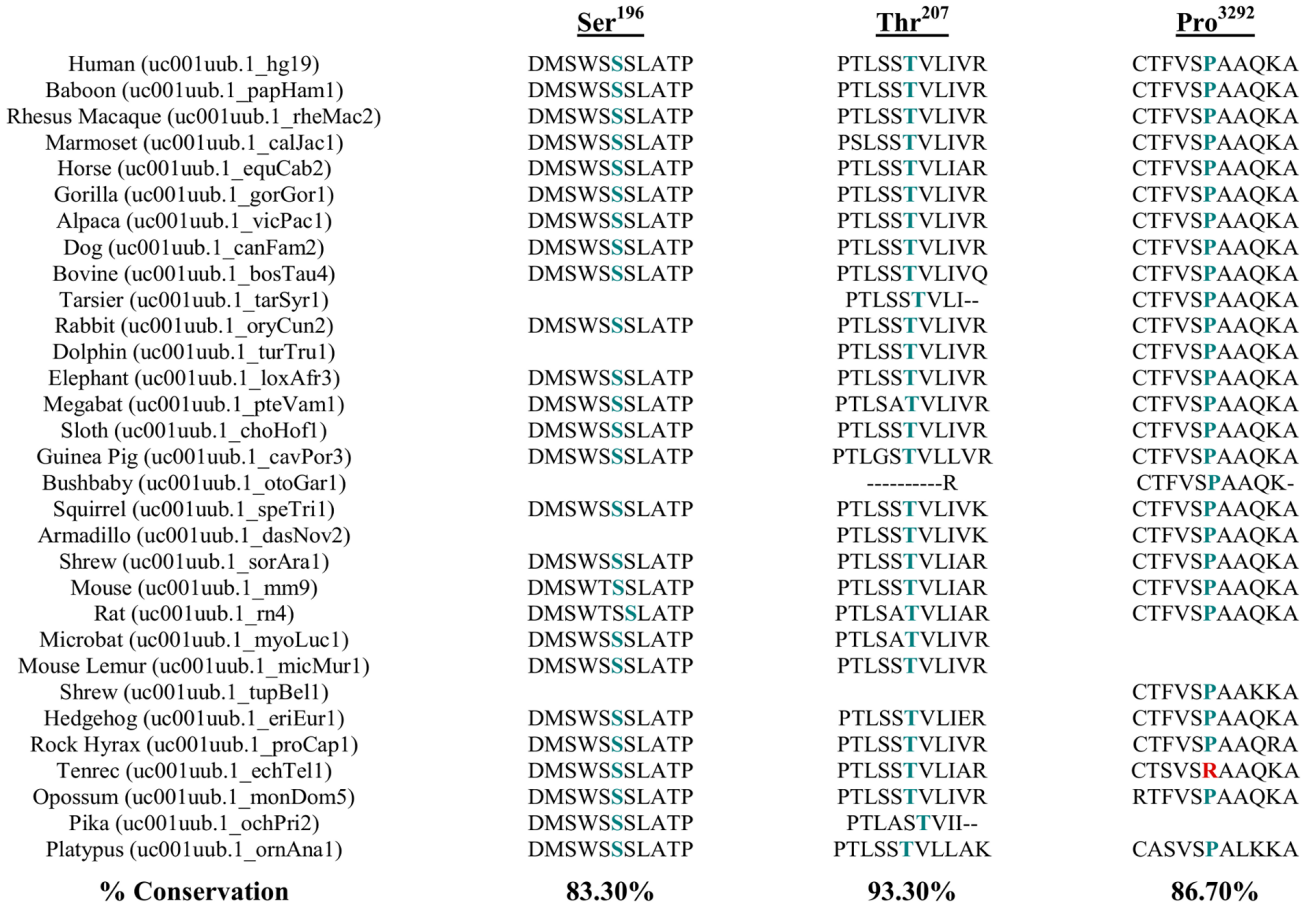
Multiple sequence alignment demonstrating phylogenetic conservation of the three biologically characterized phosphorylated BRCA2 residues affected by missense variants of unknown clinical significance.

SIFT and PolyPhen analyses were performed to evaluate whether the residues altered by VUS disrupting protein phosphorylation are damaging to protein function. Multiple sequence alignment retrieved from Polyphen results were also organized to visualize if the VUSs affect evolutionarily conserved residues. We also used A-GVGD to assign classes of C0 (neutral) to C65 (likely deleterious) to each variant. A-GVGD classified the 6 *BRCA1* VUS affecting biologically characterized sites as C0 or neutral while 66% (2/3) *BRCA2* VUS were designated a higher class (Table 1). On the other hand 26.3% (5/19) of *BRCA1* affecting uncharacterized sites were classified as possibly deleterious with 73.7% (14/19) and 100% (3/3) *BRCA2* variants being C0 (Table 2). Multiple sequence alignment from Polyphen demonstrated that 6 *BRCA1* VUS affecting biologically characterized sites were highly conserved (Figure 2) and the substitutions were predicted as either likely damaging or damaging to the protein function (Table 1). Of the 19 *BRCA1* VUS affecting biologically uncharacterized sites, 68.42% (13/19) were predicted to be likely damaging or damaging to protein function while 31.58% (6/19) VUS were benign (Table 2). Polyphen multiple sequence alignment results showed that the 3 *BRCA2* VUS affecting biologically characterized sites occurred at evolutionarily conserved sites and thus were damaging (Figure 3) and all *BRCA2* VUS affecting uncharacterized sites were also predicted to be damaging to protein function.

## Discussion

BRCA1 interacts with many proteins to serve its function in the cell. Protein kinases have been shown to be critical in BRCA1-phosyphorylation, where they are involved in activation or deactivation of the BRCA1 protein function including its stability, protein-interactions and sub-cellular location [34]–[36], its regulation of DNA repair [37]–[40] and its transcriptional activity [41]–[43]. The phosphorylation pattern of BRCA2 is less well known but it is shown to be essential in the regulation of BRCA2-mediated DNA recombination repair [44], [45].

In this study, we applied a prediction strategy based on the NetworKIN algorithm [26] to investigate the impact of VUS on the kinase-binding ability and phosphorylation patterns of BRCA1 and BRCA2 proteins. By targeting sites phosphorylated *in vivo* with clearly defined biological roles, NetworKIN analysis permits inference on biological and possibly clinical significance for any VUS that abolish kinase association at that residue. This is a significant advantage over predictions based on consensus sequence motifs recognized by active sites of enzymes alone. Therefore the method provides an effective way to identify VUS altering kinase association at key residues of biologically characterized phosphorylation sites and their potential impact can be inferred via validation assays in the literature. An added advantage of our approach is that NetworKIN can shed light on potential kinases that interact with phosphorylation sites confirmed to be phosphorylated *in vivo* using proteomic discovery methods but for which no additional experiments have yet been done to characterize their role in BRCA function.

### VUS impacting the phosphorylation of BRCA1 and BRCA2

The sixteen biologically characterized phosphorylation sites for BRCA1 (Table S1 in File S1) studied are involved in functions including intracellular localization [46], [47], transcription regulation [48], and cell cycle regulation [39], [49]. Phosphorylation of BRCA2, on the other hand, is pertinent in regulating of BRCA2-mediated DNA recombination repair [44], [45]. Overall 3.14% (6/191) of *BRCA1* and 6.98% (3/43) of *BRCA2* VUS studied represent variants of potentially high clinical significance because they occur only very rarely (n<2 in BIC) and are predicted to disrupt *in vivo* phosphorylated sites whose role in regulating BRCA1/2 functions have been biologically characterized. Lastly our results also suggest that VUS impacting phosphorylated sites tend to occur at evolutionarily conserved residues. Using the SIFT, Polyphen, and A-GVGD algorithms concurrently we ensured that all true positives were captured. This is important since the VUS impact *in vivo* phosphorylated sites and that the vast majority of the variants identified in this study do not fall within the functional domains of BRCA1 and BRCA2 where most pathogenic mutations to date are found.

### Candidate BRCA1/2 VUS for disease association studies

Six *BRCA1* VUS affected phosphorylation of BRCA1 at a biologically characterized site by altering the kinase motif and thus eliminating kinase binding. In particular, three of the VUS S632N, S1143F, and S1542C directly removed the S residue and completely abolished the biologically characterized phosphorylation sites at Ser^632^, Ser^1143^, and Ser^1542^, respectively. Although the remaining three VUS (K309T, Q1144H, Q1281P) did not directly impact the phosphorylated residue, they were predicted to alter the consensus kinase binding motif, resulting in the abolition of a phosphorylation site. For *BRCA2*, S196I, T207A, and P3292L affected phosphorylation of previously biologically characterized phosphorylation sites at Ser^193^, Thr^207^, and Ser^3291^, respectively. Given that the biological function of the affected phosphorylation sites are known, these *BRCA1* and *BRCA2* VUS are excellent candidates for further association studies into pathogenicity. In the following section, we discuss the potential biological consequences of these VUSs based on studies demonstrating their functions.

### BRCA1-K309T promotes aberrant chromosome segregation

Aurora-A/STK6 localizes to the centrosome in the G_2_-M phase, and its kinase activity positively regulates the G_2_ to M transition of the cell cycle [50]. It physically binds to and phosphorylates BRCA1 *in vivo* at Ser^308^ and that this interaction is required for the regulation of progression from G_2_ to M transition. As it has been shown that centrosome maturation from late S to M phase is essential in the completion of mitosis [51] and that Aurora-A has a role in inhibiting BRCA1-mediated centrosome nucleation in the late G_2_-M phase [52], the K309T VUS identified in breast cancer patients is a candidate mutation that may promote aberrant chromosome segregation resulting in multi-nucleation and multi-centrosomes often associated with breast cancers [53], [54].

### BRCA1-S632N affects BRCA1-mediated transcription

*In vivo* phosphorylation of BRCA1 at Ser^632^ by cyclin D1/cdk4 complex has been shown by Kehn et al [48] to inhibit DNA binding activity of BRCA1 to gene promoters during G_0_–G_1_ phase of the cell cycle. Among these gene promoters are those involved in tumor suppression (*RYBP, APEX, SST, OAS1*) as well as oncogenes involved in positively aiding tumor progression (*ARGH, FHX*). All three VUSs S632N, P633T and P633S abolished the CDK2 kinase binding at Ser^632^, but in the case of the latter two, NetworKIN predicted CDK2 binding ability at the altered residues created by threonine and serine, respectively, suggesting that only S632N completely abolishes kinase binding and thus represent a potentially pathogenic VUS due to disruption in BRCA1-mediated gene transcription.

### BRCA1-S1143F, Q1144H and Q1281P interfere with BRCA1-mediated single strand repair

Phosphorylation of Ser^1143^ and Ser^1280^ play a role in single strand break (SSB) DNA repair following alkylating agent methyl methanethiosulfonate (MMTS) exposure by contributing to the localization of BRCA1 to nuclear foci [46]. The authors showed that site-directed mutagenesis of Ser^1143^ and Ser^1280^ reduced the targeting of BRCA1 to MMTS-induced foci. Indeed, our results showing three VUS, S1143F, Q1144H and Q1281P, completely abolished ATM binding to Ser^1143^ and Ser^1280^, suggesting these are likely to contribute to the tumorigenic process by interfering with BRCA1-mediated SSB DNA repair.

### BRCA1-S1542C deregulates BRCA1-mediated double stranded break repair

ATM phosphorylates BRCA1 at Ser^1542^ *in vivo* in response to double stranded breaks (DSB) induced by γ irradiation [49], [55]. While it is unknown how phosphorylation at this site contributes to BRCA1 function, Cortez et al. demonstrated that site-directed mutagenesis of two of the seven sites (Ser^1423^ and Ser^1524^) identified from the same study were significantly more sensitive to growth inhibition by ionizing radiation compared to wildtype BRCA1 owing to the altered function of BRCA1 in post-exposure cell proliferation and recovery processes. It should be noted that while NetworKIN predicted CSNK2A2 and CK2A1 binding rather than ATM for Ser^1542^ this may be explained by the fact that in contrast to Ser^1423^ and Ser^1524^, Ser^1542^ along with four other sites identified in the study (Ser^1189^, Ser^1330^, Ser^1457^, Ser^1466^) were phosphorylated only when kinase reaction was allowed to proceed longer with higher concentrations of adenosine triphosphate and ATM [49]. Nevertheless NetworKIN found that ATM was the predicted kinase for three of the four sites (Table S1 in File S1). This suggests that ATM is the most likely kinase for Ser^1542^ and that double-strand break DNA repair following ionizing radiation may be compromised by this VUS.

### BRCA2-S196I and T207A disrupt interaction with P/CAF

Phosphorylation of highly conserved Ser^193^ and/or several Ser/Thr residues between codons 203–207 by the polo-like 1 (Plk1) kinase modulates BRCA2 disassociation from the p300/CBP-associated factor (P/CAF) [56]. Interestingly, while PLK1 was not the predicted kinase for these sites, S196I and T207A VUSs nevertheless alter highly conserved residues to deleteriously affect the consensus phosphorylation motifs of Ser^193^ and Thr^207^, respectively, to abolish kinase binding suggesting a potential link between mutations and disruption of the interaction with P/CAF.

### BRCA2-P3292L affects interaction with RAD51

BRCA2 Ser^3291^, the most well characterized phosphorylation site for BRCA2 located at the carboxy-terminal region, interacts with the recombination protein RAD51 [57]. It has been shown that phosphorylation of Ser^3291^ by CDKs blocks interaction between BRCA2 and RAD51 serving as a molecular switch for the regulation of recombination activity [44]. P3292L occurs at a highly conserved residue and abolishes CDK2 binding to Ser^3291^. This strongly suggests that this VUS is of high clinical significance and impact breast cancer by negatively affecting the interaction between BRCA2 and RAD51.

### Candidate VUS for BRCA1/2 functional studies

In this study we have also identified 19 BRCA1 and 3 BRCA2 VUS (Table 2) that were predicted to alter known *in vitro* and *in vivo* phosphorylated sites, however, not yet characterized for their biological role in protein function or in breast cancer development. Overall, our findings indicated casein kinase II (CK2) and ATM to be important kinases that bind to many biologically uncharacterized but phosphorylated sites that are affected by VUS as discussed below.

Casein Kinase II (CK2) is a ubiquitous protein serine/threonine kinase involved in SSB repair of chromosomal DNA [58]. It was first described to bind and phosphorylate the carboxyl region of BRCA1 (amino acids between 1345–1863) at Ser^1572^ [59]. In cell cycle regulation it is required in the transition from G0 to G1 and G1 to S [60]. NetworKIN prediction showed that the predicted kinase for the biologically uncharacterized sites Ser^403^, Ser^454^, Ser^749^, Ser^1214^, Ser^1217^, Ser^1218^, and Ser^1577^ to be CK2 and CSNK2A1. In support of the functional significance of this observation, four of the five *BRCA1* VUS (S454N, S1217P, S1218C and S1577P) which directly mutated serine residues at Ser^454^, Ser^1217^, Ser^1218^, and Ser^1577^ are predicted to abrogate CK2/CSNK2A1 binding to these sites. In fact 35% (7/20) *BRCA1* VUS (S403F, S454N, D749Y, E1214K, S1217P, S1218C and S1577P) are predicted to result in the abrogation of CK2A1 and CSNK2A1 interaction on these sites while N417S and P1502S created a binding site for these two kinases at Ser^417^ and Ser^1502^, respectively.

These variants likely play a role in breast cancer predisposition by deleteriously affecting BRCA1-mediated cell cycle regulation and thus warrant further investigation. Interestingly in BRCA2, the biologically uncharacterized sites Ser^1923^ and Thr^3193^ identified from a general mass spectrometry screen in prostate cancer cells [61] and non-small cell lung cancer from the CST research group [62]–[64] are also predicted to be phosphorylated by the CK2 kinases. Two of the three *BRCA2* VUSs (D1923V and D1923A), were predicted to abolish the CK2 kinase binding at Ser^1923^ which is a highly evolutionarily conserved residue, also making these variants valid targets for functional analyses in breast cancer.

Several phosphorylation sites were identified via mass spectrometry to detect phosphorylation in response to DNA damage [55], [65]–[67]. Thr^1700^ and Thr^1720^ were identified from an ATM/ATR kinase analysis and NetworKIN also predicted ATM to be the kinase for Thr1720. Thr^1700^ in the C-terminal BRCT domain of BRCA1 is part of a hydrogen bonding network with the DNA helicase BACH1 and DNA resectioning factor CtIP [68], [69] and our results show that VUSs (F1695L, R1699L) and R1699W reduce the consensus motif of Thr^1700^ to abolish the majority of kinase affinity. Interestingly R1699W is a variant known to be clinically significant as it reduces peptide binding to the pSer-x-x-Phe motifs in partner proteins that regulates the response to DNA damage [12]. These results suggest that a significant change in phosphorylation pattern of Thr^1700^ may also contribute to their clinical significance by altering the DNA damage response of BRCA1.

T1720A was the subject of several analyses including structural [70], [71], transcription [11], transactivation [71] and phospho-peptide binding assays [70] because it was the sole BRCA1 alteration in individuals considered to be at high risk for breast or ovarian cancer. These analyses suggested T1720A to be of neutral/low clinical significance. In our study, however, NetworKIN predicted ATM binding to this site, which was removed by T1720A, therefore warrants further attention with respect to kinase recognition and binding.

## Future Studies

*In silico* analysis greatly enhance our ability to make predictions on genetic variations for which currently no experimental evaluation is available. *BRCA1* and *BRCA2* variations found to affect kinase binding to these sites will be invaluable in the prioritization for further functional characterization and/or association studies in breast cancer. A follow-up study covering more comprehensive list of VUS compiled from various databases and literature sources will be a great value for the clinical management of disease in the families carrying them.

## Conclusion

The results of this study suggest for the first time that missense VUS can influence the phosphorylation patterns of BRCA1 and BRCA2. The variants identified using *in silico* methods here are based on *in vivo* phosphorylated sites and the functional evidence for the corresponding observation were also supported by the literature. Therefore the VUSs highlighted in this study are key candidate mutations that alter phosphorylated motifs to prevent kinase interactions essential for the biological functions of BRCA1 and BRCA2, and represent important candidates for further analysis into disease susceptibility. Our approach and data provide novel insights into how mutations can alter the function of BRCA1 and BRCA2 through post-translational modifications such as phosphorylation. As new phosphorylation sites are identified and their kinase specificities and biological role are elucidated, it is likely that missense variants affecting this important process will significantly contribute to the clinical management of breast cancer.

## Supporting Information

File S1 **Table** **S1,** Summary of the BRCA1 phosphorylation motifs studied. A list of all BRCA1 phosphorylation sites studied. Bolded phosphorylation site represents *in vivo* phosphorylated residues. *STK6 score fell below the cut-off value of 5 but since it has previously been shown experimentally (Ouchi, et al., 2004) it is included. ** S405 and S1286 were excluded from the study due to wildtype predictions below the score of 5. **Table** **S2,** Summary of the BRCA2 phosphorylation motifs studied. A list of all BRCA2 phosphorylation sites studied. Bolded phosphorylation site represents *in vivo* phosphorylated residues. * S206, S384, Y3009 were excluded from the study due to wildtype predictions below the score of 5. **Table** **S3,** *BRCA1* and *BRCA2* variants identified in this study to affect biologically characterized phosphorylation sites and were also previously reported in other publications (retrieved from the Leiden Open Variation Database 2.0 (Build 35)). **Table** **S4,** *BRCA1* and *BRCA2* variants identified in this study to affect biologically uncharacterized phosphorylation sites and were also previously reported in other publications (retrieved from the Leiden Open Variation Database 2.0 (Build 35)).
(DOCX)
