# Supplementary material for: Missense Variants of Uncertain Significance (VUS) Altering the Phosphorylation Patterns of BRCA1 and BRCA2
Source: PLoS One. 2013 May 21;8(5):e62468. doi: 10.1371/journal.pone.0062468 (PMC3660339; doi:10.1371/journal.pone.0062468)
Supplement: File S1 — Table S1, Summary of the BRCA1 phosphorylation motifs studied. A list of all BRCA1 phosphorylation sites studied. Bolded phosphorylation site represents in vivo phosphorylated residues. *STK6 score fell below the cut-off value of 5 but since it has previously been shown experimentally (Ouchi, et al., 2004) it is included. ** S405 and S1286 were excluded from the study due to wildtype predictions below the score of 5. Table S2, Summary of the BRCA2 phosphorylation motifs studied. A list of all BRCA2 phosphorylation sites studied. Bolded phosphorylation site represents in vivo phosphorylated residues. * S206, S384, Y3009 were excluded from the study due to wildtype predictions below the score of 5. Table S3, BRCA1 and BRCA2 variants identified in this study to affect biologically characterized phosphorylation sites and were also previously reported in other publications (retrieved from the Leiden Open Variation Database 2.0 (Build 35)). Table S4, BRCA1 and BRCA2 variants identified in this study to affect biologically uncharacterized phosphorylation sites and were also previously reported in other publications (retrieved from the Leiden Open Variation Database 2.0 (Build 35)). (DOCX) [file pone.0062468.s001.docx]

**Supporting Information**

**Legends to Supplementary Tables**

**Table S1:** Summary of the BRCA1 phosphorylation motifs studied. A list of all BRCA1 phosphorylation sites studied. Bolded phosphorylation site represents *in vivo* phosphorylated residues. *STK6 score fell below the cut-off value of 5 but since it has previously been shown experimentally (Ouchi, et al., 2004) it is included. ** S405 and S1286 were excluded from the study due to wildtype predictions below the score of 5

**Table S2:** Summary of the BRCA2 phosphorylation motifs studied. A list of all BRCA2 phosphorylation sites studied. Bolded phosphorylation site represents *in vivo* phosphorylated residues. * S206, S384, Y3009 were excluded from the study due to wildtype predictions below the score of 5.

**Table S3:** *BRCA1* and *BRCA2* variants identified in this study to affect biologically characterized phosphorylation sites and were also previously reported in other publications (retrieved from the Leiden Open Variation Database 2.0 (Build 35))

**Table S4:** *BRCA1* and *BRCA2* variants identified in this study to affect biologically uncharacterized phosphorylation sites and were also previously reported in other publications (retrieved from the Leiden Open Variation Database 2.0 (Build 35))

**Table S1**

| **Phosphorylation Sites** | **BRCA1 Function** | **Kinase (s)** | **Phoshorylation** | **BIC Missense Variants (within 10 nt of motif)** | **NetworKIN 2.0 Beta WT Output (Score>5)** |
| --- | --- | --- | --- | --- | --- |
| S114 |  |  | Biologically uncharacterized | Y105C, E111A, I124V | S114 - CDK2 - 11.9354 |
| **S308** | Cell Cycle | STK6 | Biologically characterized | E300D, K309T, R315G, S316G | *S308 - STK6 - 4.1318 |
| S395 |  |  | Biologically uncharacterized | S403F | S395 - CSNK2A2 - 18.6883 S395 - CK2A1 - 18.6883 |
| S398 |  |  | Biologically uncharacterized | S403F, K408E | S398 - CSNK2A2 - 11.7459 S398 - CK2A1 - 11.7459 S398 - TLK1 - 9.4999 |
| S403 |  |  | Biologically uncharacterized | S403F, K408E, D411N, D411E | S403 - CK2A1 - 10.5925 S403 - CSNK2A2 - 10.5925 S403 - CK2A1 - 10.5925 |
| S405** |  |  | Biologically uncharacterized | D411N, D411E | Predictions Score <5 |
| S423 |  |  | Biologically uncharacterized | N417K, N417S, D420Y, E421K, H437P | S423 - CSNK2A2 - 17.8448 S423 - CK2A1 - 17.8448 |
| S451 |  |  | Biologically uncharacterized | E445Q, S454N, I456T, F461L | S451 - TGFBR2 - 7.4107 S451 - ACVR2B - 7.3511 |
| S454 |  |  | Biologically uncharacterized | S454N, I456T, F461L, G462R | S454 - CSNK2A2 - 15.1914 S454 - CK2A1 - 15.1914 |
| **T509** | Transcription, Intracellular Localization | PKB Group | Biologically characterized | K503R, R504H, R504C, R507I, L512F | T509 - PIM2 - 14.0558 T509 - RPS6KP1 - 12.3565 |
| S510 |  |  | Biologically uncharacterized | K503R, R504H, R504C, R507I, L512F | S510 - Pim2 - 11.863 S510 - MOK - 8.9448 |
| S615 |  |  | Biologically uncharacterized | N609S, R612G, A622V | S615 - RP26KB1 - 11.6281 |
| S616 |  |  | Biologically uncharacterized | N609S, R612G, A622V, E624K | S616 - CLK1 - 7.4065 S616 - CLK2 - 7.3988 S616 - PRKCD - 6.1543 S616 - PRKC1 - 6.1357 S616 - PRKCQ - 6.1357 S616 - PRKCZ - 6.1295 S616 - PRKCA - 6.1233 S616 - PRKCG - 6.1109 S616 - RPS6KB1 - 5.7185 |
| T617 |  |  | Biologically uncharacterized | N609S, R612G, A622V, E624K, V621I | T617 - Pim2 - 11.0799 |
| **S632** | Transcription | CDK2 | Biologically characterized | A622V, E624K, V627I, S628R, S632N, P633T, P633S, P634R, P634S, E638K, D642H | S632 - CDK2 - 12.0835 |
| **S694** | Protein Stabilization |  | Biologically characterized | Q687P, D693N, D695Y, D695N, T696A | S694 - RP26KB1 - 9.3335 S694 - PIM2 - 9.1511 |
| S753 |  |  | Biologically uncharacterized | D749Y, R756S, E761A | S753 - CSNK2A2 - 6.2329 S753 - CK2A1 - 6.2329 |
| **S988** | Activation, Intracellular Localization | CHK2 | Biologically characterized | R979C, R979H | Predictions Score <5 |
| **S1143** | DNA Repair |  | Biologically characterized | P1136R, M1137T, S1139I, S1140G, A1142P, S1143F, Q1144H, C1146S, P1150S, D1152N | S1143 - ATM - 12.6322 |
| **S1189** | Molecular Association | ATM, CDK1 | Biologically characterized | V1181I, V1181A, K1183R, S1187N, T1194I, T1196K, T1196I | S1189 - CDK2 - 10.479 S1189 - MAPK14 - 8.324 S1189 - MAPK11 - 8.324 S1189 - MAPK13 - 8.299 S1189 - MAPK8 - 7.6199 S1189 - MAPK10 - 7.6123 S1189 - MAPK9 - 7.6046 |
| **S1191** | Intracellular localization | CDK1 | Biologically characterized | V1181I, V1181A, K1183R, S1187N, T1194I, T1196K, T1196I, Q1200H, Q1200H, G1201S | S1191 - MOK - 7.7417 S1191 - MAPK8 - 7.134 S1191 - MAPK10 - 7.1269 S1191 - MAPK9 - 7.1197 S1191 - MAPK8 - 7.6199 S1191 - MAPK14 - 5.8665 S1191 - MAPK11 - 5.8665 S1191 - MAPK13 - 5.8488 S1191 - CDK2 - 5.7847 S1191 - CDK5 - 5.7794 |
| S1211 |  |  | Biologically uncharacterized | G1201S, R1203Q, G1205R, K1208E, E1214K, S1218C | S1211 - CK2A1 - 20.6567 S1211 - CSNK2A2 - 20.6567 |
| S1212 |  |  | Biologically uncharacterized | R1203Q, G1205R, K1208E, E1214K, S1218C, E1219K, E1219D | S1212 - CSNK2A2 - 8.4757 S1212 - CK2A1 - 8.4757 |
| S1217 |  |  | Biologically uncharacterized | K1208E, E1214K, S1218C, E1219K, E1219D | S1217 - CSNK2A2 - 22.1941 S1217 - CK2A1 - 22.1941 |
| S1218 |  |  | Biologically uncharacterized | K1208E, E1214K, S1218C, E1219K, E1219D | S1218 - CSNK2A2 - 22.3858 S1218 - CK2A1 - 22.3858 |
| S1239 |  |  | Biologically uncharacterized | F1231L, K1233R, N1236K, I1237M, P1238R, P1238L, S1241Y, T1242A, V1247I, T1249S | S1239 - ATM - 15.4434 |
| S1245 |  |  | Biologically uncharacterized | I1237M, P1238R, P1238L, S1241Y, T1242A, V1247I, T1249S, E1250K, K1254E | S1245 - AURKB - 6.2013 S1245 - PAK4 - 5.2138 S1245 - PAK7 - 5.6122 S1245 - PAK2 - 5.1842 S1245 - PAK3 - 5.1581 |
| **S1280** | DNA Repair |  | Biologically characterized | I1275V, A1279T, Q1281P, E1282V, H1283R, H1284R | S1280 - ATM - 11.3997 |
| S1286** |  |  | Biologically uncharacterized | A1279T, Q1281P, E1282V, H1283R, H1284R, A1293D, A1293V | Predictions Score <5 |
| S1328 |  |  | Biologically uncharacterized | K1322I, D1337E | S1328 - RP26KB1 - 9.3335 S1328 - PIM2 - 9.1511 |
| S1330 |  | ATM | Biologically uncharacterized | K1322I, D1337E | S1330 - ATM - 17.5181 |
| S1336 |  |  | Biologically uncharacterized | D1337E, D1344G, E1346K | S1336 - CSNK2A2 - 18.1857 S1336 - Ck2A1 - 18.1857 |
| S1342 |  |  | Biologically uncharacterized | D1337E, D1344G, E1346K, R1347K, R1347G, T1349M, T1349P, E1352K | S1342 - CSNK2A2 - 22.2937 S1342 - CK2A1 - 22.2937 |
|  |  |  |  |  |  |
| **S1387** | Cell Cycle | ATM | Biologically characterized | T1376R, S1377R, V1378I, Q1395R | S1387 - ATM - 17.1378 |
| **S1423** | Cell Cycle, Molecular Association | ATM, ATR | Biologically characterized | E1419Q, H1421Y, H1421R, P1430S, I1432L | S1423 - ATM - 17.6874 |
| **S1457** | Molecular Association | ATM | Biologically characterized | S1448T, S1448G | S1457 - ATM - 17.8914 |
| S1466 |  | ATM | Biologically uncharacterized | N1468H, P1469S, E1470D | S1466 - ATM - 17.3447 |
| **S1497** | Molecular Association | CDK1, CDK2, ATM | Biologically characterized | K1487R, E1494K, R1495K, R1495M, P1502S, R1507T | S1497 - CDK2 - 12.6613 |
| S1499 |  |  | Biologically uncharacterized | K1487R, E1494K, R1495K, R1495M, P1502S, R1507T | GSK3B @ S1499- 5.932 GSK3A @ S1499 - 5.926 |
| **S1524** | Cell Cycle, Molecular Association | ATM, ATR | Biologically characterized | Y1522C, E1527K, I1529V, V1534M | S1524 - ATM - 16.5643 |
| **S1542** | Molecular Association | ATM | Biologically characterized | V1534M, S1542C, P1544L, D1546Y, D1546N, T1550I, L1553M | S1542 - CSNK2A2 -15.1303 S1542 - CK2A1 - 15.1303 |
| S1550 |  |  | Biologically uncharacterized | S1542C, P1544L, D1546Y, D1546N, T1550I, L1553M, D1557H, E1559K, E1559Q | T1550 - NEK2 - 6.7505 |
| **S1572** |  | CK2-A1 | Biologically characterized | T1561I, P1562L, L1564P, I1568V, P1575H, S1577P, D1578G, S1580F | T1572 - CSNK2A2 - 18.7348 T1572 - CK2A1 - 18.7348 |
| S1577 |  |  | Biologically uncharacterized | I1568V, P1575H, S1577P, D1578G, S1580F, A1584S | T1577 - CSNK2A2 - 21.2235 T1577 - CK2A1 - 21.2235 |
| T1700 |  |  | Biologically uncharacterized | T1691K, T1691I, D1692Y, D1692N, D1692H, F1695L, V1696L, C1697R, R1699Q, R1699L, R1699W, G1706A, G1706E, A1708E | T1700 - TGFBR2 - 7.3074 T1700 - ACVR2B - 7.2486 T1700 - PRKD1 - 6.6872 T1700 - PRKCD - 5.5359 T1700 - PRKCI - 5.5191 T1700 - PRKCQ - 5.5191 T1700 - PRKCZ - 5.5136 T1700 - PRKCA - 5.508 T1700 - PRKCG - 5.4968 T1700 - MST2 - 5.4712 |
| T1720 |  | ATM | Biologically uncharacterized | V1713A, V1714G, S1715C, S1715N, S1715R, W1718C, W1718S, T1720A, S1722F, R1726G, N1730S | T1720 - ATM - 12.7702 |
| **Total 44** |  |  | **16 biologically characterized 28 biologically uncharacterized sites** | **Total 191 VUS** |  |

**Table S2**

| **Phosphorylation Sites** | **BRCA2 Function** | **Kinase (s)** | **Phoshorylation** | **BIC Missense Variants (within 10 nt of motif)** | **NetworKIN 2.0 Beta WT Output (Score>5)** |
| --- | --- | --- | --- | --- | --- |
| S193 | Cell cycle | PLK1 | Biologically characterized | L184P, E187K, S196I, S196N | S193 - TGFBR2 - 5.2449 S193 - ACVR2B - 5.2026 |
| T203 | Cell cycle | PLK1 | Biologically characterized | S196I, S196N, T207A, V208G, V211L, V211I | S203 - MAP4K4 - 5.0961 S203 - TNIK - 5.0543 |
| S205 | Cell cycle | PLK1 | Biologically characterized | S196I, S196N, T207A, V208G, V211L, V211I | S205 - NEK2 - 5.3174 |
| S206* | Cell cycle | PLK1 | Biologically characterized | S196I, S196N, T207A, V208G, V211L, V211I | Predictions Score <5 |
| T207 | Cell cycle | PLK1 | Biologically characterized | T207A, V208G, V211L, V211I | T207 - NEK2 - 6.0389 |
| S239 | Cell cycle | PLK1 | Biologically characterized | H236R, D244N, I247T, A248T | S239 - TGFBR2 - 6.2295 S239 - ACVR2B - 6.1794 S239 - PRKCD - 6.1024 S239 - PRKCI - 6.0839 S239 - PRKCQ - 6.0839 S239 - PRKCZ - 6.0777 S239 - PRKCA - 6.0716 S239 - PRKCG - 6.0593 |
| S384* |  |  | Biologically uncharacterized | P375S, P375L, F376C, S384F | Predictions Score <5 |
| S683 |  |  | Biologically uncharacterized | T675R | S683 - ATM - 17.4843 |
| S755 |  |  | Biologically uncharacterized | P375S, P375L, F376C, S384F | S755 - ATM - 15.9114 |
| S1926 |  |  | Biologically uncharacterized | H1918Y, H1918R, D1923A, D1923V, E1928K, I1929V, N1935S | S1926 - CSNK2A2 - 8.8398 S1926 - CK2A1 - 8.8398 |
| S1943 |  |  | Biologically uncharacterized | N1935S , S1946P, P1947S, V1950I | Predictions Score <5 |
| Y3009* |  |  | Biologically uncharacterized | E3002K , E3002D, L3011P, T3013I | Predictions Score <5 |
| T3193 |  |  | Biologically uncharacterized | D3188N , P3194Q, T3195A, K3196E, C3198R | T3193 - CDK2 - 13.8792 |
| S3291 | Cell cycle | CDK, ATM | Biologically characterized | P3292L, A3297G | S3291 - CDK2 - 11.3492 S3291 - MAPK11 - 8.4881 S3291 - MAPK13 - 8.4625 S3291 - MAPK14 - 8.4881 |
| **Total 11** |  |  | **7 Biologically characterized 4 Biologically uncharacterized** | **Total 43 VUS** |  |

**Table S3**

| **Gene** | **Exon** | **Codon** | **Variant as Published** | **DNA Change** | **Protein** | **Reference** | **Assay** | **Assay Result** |
| --- | --- | --- | --- | --- | --- | --- | --- | --- |
| BRCA1 | 11 | 632 | S632N | [c.1895G>A](http://chromium.liacs.nl/LOVD2/cancer/variants.php?select_db=BRCA1&action=view&view=0000414%2C0000414%2C0) | p.Ser632Asn | [Fleming et al. (2003)](http://www.ncbi.nlm.nih.gov/pubmed/12531920) | Evolutionary conservation analysis | Predicted Deleterious |
| BRCA1 | 11 | 632 | S632N | [c.1895G>A](http://chromium.liacs.nl/LOVD2/cancer/variants.php?select_db=BRCA1&action=view&view=0000415%2C0000415%2C0) | p.Ser632Asn | [Burk-Herrick et al. (2006)](http://www.ncbi.nlm.nih.gov/pubmed/16518693) | Evolutionary conservation analysis | Inconclusive |
| BRCA1 | 11 | 632 | S632N | [c.1895G>A](http://chromium.liacs.nl/LOVD2/cancer/variants.php?select_db=BRCA1&action=view&view=0000416%2C0000416%2C0) | p.Ser632Asn | [Abkevich et al. (2004)](http://www.ncbi.nlm.nih.gov/pubmed/15235020) | Evolutionary conservation analysis | Predicted Neutral |
| BRCA1 | 11 | 1144 | Q1144H | [c.3432G>T](http://chromium.liacs.nl/LOVD2/cancer/variants.php?select_db=BRCA1&action=view&view=0000638%2C0000638%2C0) | p.Glu1144His | [Burk-Herrick et al. (2006)](http://www.ncbi.nlm.nih.gov/pubmed/16518693) | Evolutionary conservation analysis | Inconclusive |
| BRCA2 | 7 | 207 | T207A | [c.619A>G](http://chromium.liacs.nl/LOVD2/cancer/variants.php?select_db=BRCA2&action=view&view=0001521%2C0000066%2C0) | p.Thr207Ala | Pettigrew et al. (2007) | Co-localization to predicted ESEs | Predicted Deleterious |

**Table S4**

| **Gene** | **Exon** | **Codon** | **Variant as Published** | **DNA Change** | **Protein** | **Reference** | **Assay** | **Assay Result** |
| --- | --- | --- | --- | --- | --- | --- | --- | --- |
| BRCA1 | 11 | 417 | N417S | [c.1250A>G](http://chromium.liacs.nl/LOVD2/cancer/variants.php?select_db=BRCA1&action=view&view=0000311%2C0000311%2C0) | p.Asn417Ser | [Abkevich et al. (2004)](http://www.ncbi.nlm.nih.gov/pubmed/15235020) | Evolutionary conservation analysis | Predicted Neurtral |
| BRCA1 | 11 | 417 | N417S | [c.1250A>G](http://chromium.liacs.nl/LOVD2/cancer/variants.php?select_db=BRCA1&action=view&view=0000312%2C0000312%2C0) | p.Asn417Ser | [Burk-Herrick et al. (2006)](http://www.ncbi.nlm.nih.gov/pubmed/16518693) | Evolutionary conservation analysis | Inconclusive |
| BRCA1 | 11 | 420 | D420Y | [c.1258G>T](http://chromium.liacs.nl/LOVD2/cancer/variants.php?select_db=BRCA1&action=view&view=0000313%2C0000313%2C0) | p.Asp420Tyr | [Easton et al. (2007)](http://www.ncbi.nlm.nih.gov/pubmed/17924331) | Multifactorial likelihood-ratio model | Predicted Neurtral |
| BRCA1 | 11 | 420 | D420Y | [c.1258G>T](http://chromium.liacs.nl/LOVD2/cancer/variants.php?select_db=BRCA1&action=view&view=0000314%2C0000314%2C0) | p.Asp420Tyr | [Burk-Herrick et al. (2006)](http://www.ncbi.nlm.nih.gov/pubmed/16518693) | Evolutionary conservation analysis | Inconclusive |
| BRCA1 | 11 | 454 | S454N | [c.1361G>A](http://chromium.liacs.nl/LOVD2/cancer/variants.php?select_db=BRCA1&action=view&view=0000318%2C0000318%2C0) | p.Ser454Asn | [Abkevich et al. (2004)](http://www.ncbi.nlm.nih.gov/pubmed/15235020) | Evolutionary conservation analysis | Predicted Neurtral |
| BRCA1 | 11 | 609 | N609S | [c.1826A>G](http://chromium.liacs.nl/LOVD2/cancer/variants.php?select_db=BRCA1&action=view&view=0000400%2C0000400%2C0) | p.Asn609Ser | [Burk-Herrick et al. (2006)](http://www.ncbi.nlm.nih.gov/pubmed/16518693) | Evolutionary conservation analysis | Inconclusive |
| BRCA1 | 11 | 612 | R612G | [c.1834A>G](http://chromium.liacs.nl/LOVD2/cancer/variants.php?select_db=BRCA1&action=view&view=0000401%2C0000401%2C0) | p.Arg612Gly | [Fleming et al. (2003)](http://www.ncbi.nlm.nih.gov/pubmed/12531920) | Evolutionary conservation analysis | Predicted Deleterious |
| BRCA1 | 11 | 612 | R612G | [c.1834A>G](http://chromium.liacs.nl/LOVD2/cancer/variants.php?select_db=BRCA1&action=view&view=0000402%2C0000402%2C0) | p.Arg612Gly | [Burk-Herrick et al. (2006)](http://www.ncbi.nlm.nih.gov/pubmed/16518693) | Evolutionary conservation analysis | Predicted Deleterious |
| BRCA1 | 11 | 612 | R612G | [c.1834A>G](http://chromium.liacs.nl/LOVD2/cancer/variants.php?select_db=BRCA1&action=view&view=0000403%2C0000403%2C0) | p.Arg612Gly | [Ramirez et al. (2004)](http://www.ncbi.nlm.nih.gov/pubmed/15001988) | Evolutionary conservation analysis | Predicted Deleterious |
| BRCA1 | 11 | 1201 | G1201S | [c.3601G>A](http://chromium.liacs.nl/LOVD2/cancer/variants.php?select_db=BRCA1&action=view&view=0000660%2C0000660%2C0) | p.Gly1201Ser | [Salazar et al. (2006)](http://www.ncbi.nlm.nih.gov/pubmed/15876480) | N/A | N/A |
| BRCA1 | 11 | 1214 | E1214K | [c.3640G>A](http://chromium.liacs.nl/LOVD2/cancer/variants.php?select_db=BRCA1&action=view&view=0000664%2C0000664%2C0) | p.Glu1214Lys | [Easton et al. (2007)](http://www.ncbi.nlm.nih.gov/pubmed/17924331) | Multifactorial likelihood-ratio model | Predicted Neurtral |
| BRCA1 | 11 | 1214 | E1214K | [c.3640G>A](http://chromium.liacs.nl/LOVD2/cancer/variants.php?select_db=BRCA1&action=view&view=0000665%2C0000665%2C0) | p.Glu1214Lys | [Burk-Herrick et al. (2006)](http://www.ncbi.nlm.nih.gov/pubmed/16518693) | Evolutionary conservation analysis | Inconclusive |
| BRCA1 | 11 | 1218 | S1218C | [c.3652A>T](http://chromium.liacs.nl/LOVD2/cancer/variants.php?select_db=BRCA1&action=view&view=0000666%2C0000666%2C0) | p.Ser1218Cys | [Burk-Herrick et al. (2006)](http://www.ncbi.nlm.nih.gov/pubmed/16518693) | Evolutionary conservation analysis | Predicted Deleterious |
| BRCA1 | 18 | 1695 | F1695L | [c.5085T>A](http://chromium.liacs.nl/LOVD2/cancer/variants.php?select_db=BRCA1&action=view&view=0000949%2C0000949%2C0) | p.Phe1695Leu | [Joo et al (2002)](http://www.ncbi.nlm.nih.gov/pubmed/11877378) | Peptide binding ability | As mutant control |
| BRCA1 | 18 | 1695 | F1695L | [c.5085T>A](http://chromium.liacs.nl/LOVD2/cancer/variants.php?select_db=BRCA1&action=view&view=0000950%2C0000950%2C0) | p.Phe1695Leu | [Mirkovic et al. (2004)](http://www.ncbi.nlm.nih.gov/pubmed/15172985) | Structure-based prediction (crystallography etc) | Predicted neutral |
| BRCA1 | 18 | 1695 | F1695L | [c.5085T>A](http://chromium.liacs.nl/LOVD2/cancer/variants.php?select_db=BRCA1&action=view&view=0000951%2C0000951%2C0) | p.Phe1695Leu | [Williams et al. (2004)](http://www.ncbi.nlm.nih.gov/pubmed/15133503) | Peptide binding ability | As wildtype control |
| BRCA1 | 18 | 1695 | F1695L | [c.5085T>A](http://chromium.liacs.nl/LOVD2/cancer/variants.php?select_db=BRCA1&action=view&view=0000952%2C0000952%2C0) | p.Phe1695Leu | [Karchin et al. (2007)](http://www.ncbi.nlm.nih.gov/pubmed/17305420) | Multifactorial likelihood-ratio model | Predicted deleterious |
| BRCA1 | 18 | 1695 | F1695L | [c.5085T>A](http://chromium.liacs.nl/LOVD2/cancer/variants.php?select_db=BRCA1&action=view&view=0000953%2C0000953%2C0) | p.Phe1695Leu | [Glover et al. (2006)](http://www.ncbi.nlm.nih.gov/pubmed/16528612) | Peptide binding ability | As wildtype control |
| BRCA1 | 18 | 1695 | F1695L | [c.5085T>A](http://chromium.liacs.nl/LOVD2/cancer/variants.php?select_db=BRCA1&action=view&view=0000954%2C0000954%2C0) | p.Phe1695Leu | [Williams et al. (2003)](http://www.ncbi.nlm.nih.gov/pubmed/14534301) | Bayesian statistics | Predicted neutral |
| BRCA1 | 18 | 1695 | F1695L | [c.5085T>A](http://chromium.liacs.nl/LOVD2/cancer/variants.php?select_db=BRCA1&action=view&view=0000955%2C0000955%2C0) | p.Phe1695Leu | [Williams et al. (2003)](http://www.ncbi.nlm.nih.gov/pubmed/14534301) | Proteolytic degradation | As wildtype control |
| BRCA1 | 18 | 1695 | F1695L | [c.5085T>A](http://chromium.liacs.nl/LOVD2/cancer/variants.php?select_db=BRCA1&action=view&view=0000956%2C0000956%2C0) | p.Phe1695Leu | [Abkevich et al. (2004)](http://www.ncbi.nlm.nih.gov/pubmed/15235020) | Evolutionary conservation analysis | Predicted neutral |
| BRCA1 | 18 | 1695 | F1695L | [c.5085T>A](http://chromium.liacs.nl/LOVD2/cancer/variants.php?select_db=BRCA1&action=view&view=0002450%2C0001530%2C0) | p.Phe1695Leu | [Lee et al. (2010)](http://www.ncbi.nlm.nih.gov/pubmed/20516115) | Transcription activation/PS/BA/BS | No Functional Effect |
| BRCA1 |  |  |  | + 74 others |  |  |  |  |
| BRCA1 | 18 | 1699 | R1699W | [c.5095C>T](http://chromium.liacs.nl/LOVD2/cancer/variants.php?select_db=BRCA1&action=view&view=0000977%2C0000977%2C0) | p.Arg1699Trp | [Glover et al. (2006)](http://www.ncbi.nlm.nih.gov/pubmed/16528612) | Peptide binding ability | As mutant control |
| BRCA1 | 18 | 1699 | R1699W | [c.5095C>T](http://chromium.liacs.nl/LOVD2/cancer/variants.php?select_db=BRCA1&action=view&view=0000978%2C0000978%2C0) | p.Arg1699Trp | [Mirkovic et al. (2004)](http://www.ncbi.nlm.nih.gov/pubmed/15172985) | Structure-based prediction (crystallography etc) | Predicted deleterious |
| BRCA1 | 18 | 1699 | R1699W | [c.5095C>T](http://chromium.liacs.nl/LOVD2/cancer/variants.php?select_db=BRCA1&action=view&view=0000979%2C0000979%2C0) | p.Arg1699Trp | [Nikolopoulos et al. (2007)](http://www.ncbi.nlm.nih.gov/pubmed/17493881) | Peptide binding ability | As mutant control |
| BRCA1 | 18 | 1699 | R1699W | [c.5095C>T](http://chromium.liacs.nl/LOVD2/cancer/variants.php?select_db=BRCA1&action=view&view=0000980%2C0000980%2C0) | p.Arg1699Trp | [Williams et al. (2003)](http://www.ncbi.nlm.nih.gov/pubmed/14534301) | Proteolytic degradation | Inconclusive |
| BRCA1 | 18 | 1699 | R1699W | [c.5095C>T](http://chromium.liacs.nl/LOVD2/cancer/variants.php?select_db=BRCA1&action=view&view=0000981%2C0000981%2C0) | p.Arg1699Trp | [Williams et al. (2003)](http://www.ncbi.nlm.nih.gov/pubmed/14534301) | Bayesian statistics | Predicted deleterious |
| BRCA1 | 18 | 1699 | R1699W | [c.5095C>T](http://chromium.liacs.nl/LOVD2/cancer/variants.php?select_db=BRCA1&action=view&view=0000982%2C0000982%2C0) | p.Arg1699Trp | [Nikolopoulos et al. (2007)](http://www.ncbi.nlm.nih.gov/pubmed/17493881) | Thermostability assay | As mutant control |
| BRCA1 | 18 | 1699 | R1699W | [c.5095C>T](http://chromium.liacs.nl/LOVD2/cancer/variants.php?select_db=BRCA1&action=view&view=0000983%2C0000983%2C0) | p.Arg1699Trp | [Shiozaki et al. (2004)](http://www.ncbi.nlm.nih.gov/pubmed/15125843) | Peptide binding ability | Predicted deleterious |
| BRCA1 | 18 | 1699 | R1699W | [c.5095C>T](http://chromium.liacs.nl/LOVD2/cancer/variants.php?select_db=BRCA1&action=view&view=0000984%2C0000984%2C0) | p.Arg1699Trp | [Worley et al. (2002)](http://www.ncbi.nlm.nih.gov/pubmed/12496476) | Transcription activation in human cells | As mutant control |
| BRCA1 | 18 | 1699 | R1699W | [c.5095C>T](http://chromium.liacs.nl/LOVD2/cancer/variants.php?select_db=BRCA1&action=view&view=0000985%2C0000985%2C0) | p.Arg1699Trp | [Gomez-Garcia et al. (2009)](http://www.ncbi.nlm.nih.gov/pubmed/19150617) | Family history | Predicted deleterious |
| BRCA1 | 18 | 1699 | R1699W | [c.5095C>T](http://chromium.liacs.nl/LOVD2/cancer/variants.php?select_db=BRCA1&action=view&view=0000986%2C0000986%2C0) | p.Arg1699Trp | [Clapperton et al. (2004)](http://www.ncbi.nlm.nih.gov/pubmed/15133502) | N/A | N/A |
| BRCA1 | 18 | 1699 | R1699W | [c.5095C>T](http://chromium.liacs.nl/LOVD2/cancer/variants.php?select_db=BRCA1&action=view&view=0000987%2C0000987%2C0) | p.Arg1699Trp | [Osorio et al. (2007)](http://www.ncbi.nlm.nih.gov/pubmed/17279547) | Multifactorial likelihood-ratio model | Predicted deleterious |
| BRCA1 | 18 | 1699 | R1699W | [c.5095C>T](http://chromium.liacs.nl/LOVD2/cancer/variants.php?select_db=BRCA1&action=view&view=0000988%2C0000988%2C0) | p.Arg1699Trp | [Williams et al. (2004)](http://www.ncbi.nlm.nih.gov/pubmed/15133503) | Peptide binding ability | As mutant control |
| BRCA1 | 18 | 1699 | R1699W | [c.5095C>T](http://chromium.liacs.nl/LOVD2/cancer/variants.php?select_db=BRCA1&action=view&view=0000989%2C0000989%2C0) | p.Arg1699Trp | [Easton et al. (2007)](http://www.ncbi.nlm.nih.gov/pubmed/17924331) | Multifactorial likelihood-ratio model | Predicted deleterious |
| BRCA1 | 18 | 1699 | R1699W | [c.5095C>T](http://chromium.liacs.nl/LOVD2/cancer/variants.php?select_db=BRCA1&action=view&view=0000990%2C0000990%2C0) | p.Arg1699Trp | [Williams et al. (2001)](http://www.ncbi.nlm.nih.gov/pubmed/11573086) | Structure-based prediction (crystallography etc) | Predicted deleterious |
| BRCA1 | 18 | 1699 | R1699W | [c.5095C>T](http://chromium.liacs.nl/LOVD2/cancer/variants.php?select_db=BRCA1&action=view&view=0000991%2C0000991%2C0) | p.Arg1699Trp | [Vallon-Christersson et al. (2001)](http://www.ncbi.nlm.nih.gov/pubmed/11157798) | Transcription activation in yeast (GAL4-fusions) | As wildtype control |
| BRCA1 | 18 | 1699 | R1699W | [c.5095C>T](http://chromium.liacs.nl/LOVD2/cancer/variants.php?select_db=BRCA1&action=view&view=0000992%2C0000992%2C0) | p.Arg1699Trp | [Worley et al. (2002)](http://www.ncbi.nlm.nih.gov/pubmed/12496476) | Transcription activation in yeast (GAL4-fusions) | As mutant control |
| BRCA1 | 18 | 1699 | R1699W | [c.5095C>T](http://chromium.liacs.nl/LOVD2/cancer/variants.php?select_db=BRCA1&action=view&view=0000993%2C0000993%2C0) | p.Arg1699Trp | [Varma et al. (2005)](http://www.ncbi.nlm.nih.gov/pubmed/16101277) | Structure-based prediction (crystallography etc) | Predicted deleterious |
| BRCA1 | 18 | 1699 | R1699W | [c.5095C>T](http://chromium.liacs.nl/LOVD2/cancer/variants.php?select_db=BRCA1&action=view&view=0002450%2C0001533%2C0) | p.Arg1699Trp | [Lee et al. (2010)](http://www.ncbi.nlm.nih.gov/pubmed/20516115) | Transcription activation/PS/BA/BS | Strong functional effect |
| BRCA1 |  |  |  | + 74 others |  |  |  |  |
| BRCA1 | 18 | 1699 | R1699W | [c.5095C>T](http://chromium.liacs.nl/LOVD2/cancer/variants.php?select_db=BRCA1&action=view&view=0002480%2C0001651%2C0) | p.Arg1699Trp | [Rowling et al. (2010)](http://www.ncbi.nlm.nih.gov/pubmed/20378548) | Thermodynamic stability prediction | Moderately Destabilizing |
| BRCA1 | 18 | 1699 | R1699L | [c.5096G>T](http://chromium.liacs.nl/LOVD2/cancer/variants.php?select_db=BRCA1&action=view&view=0000994%2C0000994%2C0) | p.Arg1699Leu | [Pettigrew et al. (2005)](http://www.ncbi.nlm.nih.gov/pubmed/16280041) | Evolutionary conservation analysis | Predicted deleterious |
| BRCA1 | 18 | 1699 | R1699L | [c.5096G>T](http://chromium.liacs.nl/LOVD2/cancer/variants.php?select_db=BRCA1&action=view&view=0000995%2C0000995%2C0) | p.Arg1699Leu | [Abkevich et al. (2004)](http://www.ncbi.nlm.nih.gov/pubmed/15235020) | Evolutionary conservation analysis | Predicted deleterious |
| BRCA1 | 18 | 1699 | R1699L | [c.5096G>T](http://chromium.liacs.nl/LOVD2/cancer/variants.php?select_db=BRCA1&action=view&view=0000996%2C0000996%2C0) | p.Arg1699Leu | [Williams et al. (2001)](http://www.ncbi.nlm.nih.gov/pubmed/11573086) | Structure-based prediction (crystallography etc) | Predicted deleterious |
| BRCA1 | 18 | 1699 | R1699L | [c.5096G>T](http://chromium.liacs.nl/LOVD2/cancer/variants.php?select_db=BRCA1&action=view&view=0000997%2C0000997%2C0) | p.Arg1699Leu | [Mirkovic et al. (2004)](http://www.ncbi.nlm.nih.gov/pubmed/15172985) | Structure-based prediction (crystallography etc) | Predicted deleterious |
| BRCA1 | 18 | 1699 | R1699L | [c.5096G>T](http://chromium.liacs.nl/LOVD2/cancer/variants.php?select_db=BRCA1&action=view&view=0002450%2C0001535%2C0) | p.Arg1699Leu | [Lee et al. (2010)](http://www.ncbi.nlm.nih.gov/pubmed/20516115) | Transcription activation/PS/BA/BS | Moderate functional effect |
| BRCA1 |  |  |  | + 74 others |  |  |  |  |
| BRCA1 | 18 | 1699 | R1699L | [c.5096G>T](http://chromium.liacs.nl/LOVD2/cancer/variants.php?select_db=BRCA1&action=view&view=0002468%2C0001639%2C0) | p.Arg1699Leu | [Rowling et al. (2010)](http://www.ncbi.nlm.nih.gov/pubmed/20378548) | Thermodynamic stability prediction | not destabilizing |
| BRCA1 | 19 | 1720 | T1720A | [c.5158A>G](http://chromium.liacs.nl/LOVD2/cancer/variants.php?select_db=BRCA1&action=view&view=0001111%2C0001111%2C0) | p.Thr1720Ala | [Williams et al. (2003)](http://www.ncbi.nlm.nih.gov/pubmed/14534301) | Proteolytic degradation | As wildtype control |
| BRCA1 | 19 | 1720 | T1720A | [c.5158A>G](http://chromium.liacs.nl/LOVD2/cancer/variants.php?select_db=BRCA1&action=view&view=0001112%2C0001112%2C0) | p.Thr1720Ala | [Williams et al. (2003)](http://www.ncbi.nlm.nih.gov/pubmed/14534301) | Bayesian statistics | Predicted neutral |
| BRCA1 | 19 | 1720 | T1720A | [c.5158A>G](http://chromium.liacs.nl/LOVD2/cancer/variants.php?select_db=BRCA1&action=view&view=0001113%2C0001113%2C0) | p.Thr1720Ala | [Glover et al. (2006)](http://www.ncbi.nlm.nih.gov/pubmed/16528612) | Peptide binding ability | As wildtype control |
| BRCA1 | 19 | 1720 | T1720A | [c.5158A>G](http://chromium.liacs.nl/LOVD2/cancer/variants.php?select_db=BRCA1&action=view&view=0001114%2C0001114%2C0) | p.Thr1720Ala | [Phelan et al (2005)](http://www.ncbi.nlm.nih.gov/pubmed/15689452) | Transcription activation in yeast (GAL4-fusions) | Inconclusive |
| BRCA1 | 19 | 1720 | T1720A | [c.5158A>G](http://chromium.liacs.nl/LOVD2/cancer/variants.php?select_db=BRCA1&action=view&view=0001115%2C0001115%2C0) | p.Thr1720Ala | [Mirkovic et al. (2004)](http://www.ncbi.nlm.nih.gov/pubmed/15172985) | Structure-based prediction (crystallography etc) | Predicted neutral |
| BRCA1 | 19 | 1720 | T1720A | [c.5158A>G](http://chromium.liacs.nl/LOVD2/cancer/variants.php?select_db=BRCA1&action=view&view=0001116%2C0001116%2C0) | p.Thr1720Ala | [McKean-Cowdin et al. (2005)](http://www.ncbi.nlm.nih.gov/pubmed/15726418) | N/A | N/A |
| BRCA1 | 19 | 1720 | T1720A | [c.5158A>G](http://chromium.liacs.nl/LOVD2/cancer/variants.php?select_db=BRCA1&action=view&view=0001117%2C0001117%2C0) | p.Thr1720Ala | [Diez et al. (2003)](http://www.ncbi.nlm.nih.gov/pubmed/12955716) | N/A | N/A |
| BRCA1 | 19 | 1720 | T1720A | [c.5158A>G](http://chromium.liacs.nl/LOVD2/cancer/variants.php?select_db=BRCA1&action=view&view=0001118%2C0001118%2C0) | p.Thr1720Ala | [Williams et al. (2004)](http://www.ncbi.nlm.nih.gov/pubmed/15133503) | Peptide binding ability | As wildtype control |
| BRCA1 | 19 | 1720 | T1720A | [c.5158A>G](http://chromium.liacs.nl/LOVD2/cancer/variants.php?select_db=BRCA1&action=view&view=0001119%2C0001119%2C0) | p.Thr1720Ala | [Easton et al. (2007)](http://www.ncbi.nlm.nih.gov/pubmed/17924331) | Multifactorial likelihood-ratio model | Predicted neutral |
| BRCA1 | 19 | 1720 | T1720A | [c.5158A>G](http://chromium.liacs.nl/LOVD2/cancer/variants.php?select_db=BRCA1&action=view&view=0002450%2C0001548%2C0) | p.Thr1720Ala | [Lee et al. (2010)](http://www.ncbi.nlm.nih.gov/pubmed/20516115) | Transcription activation/PS/BA/BS | No Functional Effect |
|  |  |  |  | + 74 others |  |  |  |  |
